# Supplementary material for: Comparison of Mitochondrial Genomes between a Cytoplasmic Male-Sterile Line and Its Restorer Line for Identifying Candidate CMS Genes in Gossypium hirsutum
Source: Int J Mol Sci. 2022 Aug 16;23(16):9198. doi: 10.3390/ijms23169198 (PMC9409232; doi:10.3390/ijms23169198)
Supplement: Supplementary file 1 [file ijms-23-09198-s001.zip › ijms-1825514-supplementary.pdf]

Table S1 the percentage of nuclear and cytoplasmic reads in Illumina and Nanopore sequencing data

**Sequencing platform**	**Short-reads sequencing (Illumina)**		
Sample	Map to nuclear genome	Map to cytoplasmic genome (in theory)	Map to mitochondria genome
0-613-2R	95.21%	4.79%	2.27%
SI3A	96.42%	3.58%	1.42%
**Sequencing platform**	**Long-reads sequencing (Nanopore)**		
Sample	Map to nuclear genome	Map to cytoplasmic genome (in theory)	Map to mitochondria genome
0-613-2R	96.08%	3.92%	2.57%
SI3A	94.98%	5.02%	2.70%

All proportions are calculated using reads with exact alignment ignoring supplementary and secondary alignment. Proportion of reads mapped to cytoplasmic genome is calculated 1 - proportion of reads mapped to nuclear genome.

Table S2 the primers of RT-PCR and qPCR for four specific ORFs.

Primer name	Forward sequence (5'-3')	Reverse sequence (5'- 3')	Use
qorf606a	TGTAGATGCTACGGGTGCTT	CTGCACGCATTCCAATACGA	To measure mitochondrial gene expression of 0-613-2R and SI3A by qRT-PCR
qorf109a	AGGGCCAGGATTCAATCTCC	ACTTGTCGGAAATCCTCGGT	
qorf131a	CGTGCAACCATCAAACCAGA	GAAATACGAACAACCGCGCT	
qorf119a-2	TGAATTTTGCTGCCAACCCA	CCGCTTCTTCCCCTTCTCTT	
RTorf606a	TACTTCAGGGGCACACACAA	TGATCCCAGCGTCTCATGAA	To assess gene expression in 0-613-2R and SI3A
RTorf109a	TCCCGGTGAAAAGAGATCCA	TCTCTATAGTTCCCCTTCTTGCT	
RTorf131a	CGTGCAACCATCAAACCAGA	ACCCAAAGCTGAACATATCCG	
RTorf119a-2	GGTCAGTCTCGCTACCTCTT	CCGCTTCTTCCCCTTCTCTT	

Table S3 Contents of CMS line SI3A and restorer line 0-613-2R.

Product group	Gene	0-613-2R	SI3Amt1	SI3Amt2	SI3Amt3	Product group	Gene	0-613-2R	SI3Amt1	SI3Amt2	SI3Amt3
complex I	nad3	+	-	+	-	Ribosome	rps7	+	-	+	-
	nad4	+	-	-	+		rps10	+	+	-	-
	nad4L	+	-	+	-		rps12	+	-	+	-
	nad6	+	-	-	+		rps14	+	-	+	-
	nad7	+	-	+	-		rpL2	+	-	+	-
	nad9	+	-	-	+		rpL5	+	-	+	-
complex II	sdh3	+	-	-	+		rpL10	+	-	+	-
	sdh4	+	+	-	-		rpL16	+	-	+	-
complex III	Cob	+	-	+	-	tRNA	trnK-UUU	+	-	+	-
complex IV	cox1	+	+	-	-		trnS-UGA	+	-	+	-
	cox2	+	-	+	-		trnfM-CAU	+4	+	+2	+
	cox3	+	+	-	-		trnM-CAU	+	+	-	-
complex V	atp1	+	+	-	-		trnY-GUA	+	-	+	-
	atp4	+	-	-	+		trnN-GUU	+	-	+	-
	atp6	+	-	+	-		trnC-GCA	+	-	+	-
	atp8	+	-	+	-		trnE-UUC	+	-	+	-
	atp9	+	-	+	-		trnH-GUG	+	-	+	-
Cytochrome C	ccmB	+	-	+	-		trnS-GCU	+2	-	+	-
	ccmC	+	-	-	+		trnF-GAA	+2	-	+	-
	ccmFN	+	-	+	-		trnP-UGG	+2	-	+2	-
	ccmFC	+	+	-	-		trnW-CCA	+2	-	+2	-
rRNA	rrn5	+2[1]	+	-	+		trnV-GAC	+	-	+	-
	rrn18	+2	+	-	+		trnD-GUC	+2	-	+	+
	rrn26	+2	+	+	-		trnS-GGA	+	-	+	-
Other gene	mttB	+	-	-	+		trnQ-UUG	+	-	-	+
	matR	+	-	+	-		trnG-GCC	+	-	-	+
Ribosome	rps3	+	-	+	-		trnM-UAU	+	-	-	+
	rps4	+	-	+	-						

+ denotes present; - denotes absent; Gene copy number is after +;

Table S4 Repeat sequences (>50bp) found in restore line 0-613-2R.

		copy1				copy2			
**No**	**Size(bp)**	**Start1**	**stop1**	**Type**	**Size(bp)**	**Start2**	**stop2**	**Identity (%)**	**Difference between copies**
R1	10151	206040	216190	DR [a]	10144	459853	469996	99	copy1 8bp indel，copy2 15bp indel.
R2	10247	23665	33911	IR [b]	10259	482599	492857	99	copy1 20bp indel, 2bp mismatch, copy2 8bp indel.
R3	2211	117463	119673	IR	2211	511704	513914	100	
R4	2019	107865	109883	IR	2019	521513	523531	100	
R5	1185	111448	112632	IR	1185	518749	519933	100	
R6	1181	114321	115501	IR	1181	515871	517051	100	
R7	1095	112831	113925	IR	1095	517459	518553	100	
R8	786	109887	110672	IR	786	520722	521507	100	
R10	517	115975	116491	IR	517	514884	515400	100	
R11	509	116720	117228	IR	509	514147	514655	100	
R12	309	114011	114319	IR	309	517060	517368	100	
R15	257	207608	207864	IR	257	508650	508906	100	
R16	257	461418	461674	IR	257	508650	508906	100	
R19	232	111093	111324	IR	232	520060	520291	100	
R20	229	58617	58845	IR	229	585876	586104	100	
R21	229	116490	116718	IR	229	514656	514884	100	
R22	219	283381	283599	IR	219	513808	514026	100	
R23	204	117245	117448	IR	204	513927	514130	100	
R25	194	311458	311651	IR	194	489100	489293	100	
R26	192	115690	115881	IR	192	515492	515683	100	
R27	175	128668	128842	IR	175	243145	243319	100	
R28	151	252393	252543	IR	151	311285	311435	100	
R29	150	112683	112832	IR	150	518553	518702	100	
R30	144	110669	110812	IR	144	520581	520724	100	
R34	118	243284	243401	IR	118	308043	308160	100	
R35	117	115576	115692	IR	117	515680	515796	100	
R36	113	16496	16608	IR	113	300055	300167	100	

R37	107	27370	27476	IR	107	236141	236247	100
R39	107	166348	166454	IR	107	544550	544656	100
R43	96	111356	111451	IR	96	519931	520026	100
R48	90	111273	111362	IR	90	458595	458684	100
R49	86	111011	111096	IR	86	520289	520374	100
R52	80	243284	243363	IR	80	495464	495543	100
R53	78	205303	205380	IR	78	414550	414627	100
R54	76	110888	110963	IR	76	520425	520500	100
R55	71	50783	50853	IR	71	270658	270728	100
R56	71	117929	117999	IR	71	232143	232213	100
R61	66	414403	414468	IR	66	455624	455689	100
R62	62	115883	115944	IR	62	515429	515490	100
R64	61	300080	300140	IR	61	386862	386922	100
R67	57	313653	313709	IR	57	437442	437498	100
R70	53	75044	75096	IR	53	290150	290202	100
R76	51	359851	359901	IR	51	456462	456512	100
R77	50	171446	171495	IR	50	204672	204721	100

[a] DR: direct repeats, [b] IR: invert repeats

Table S5 The open reading frames (ORFs) more than 100 amino acids in 0-613-2R mitochondrial genome

**Gene ID**	**strand**	**mRNA start(bp)**	**mRNA stop(bp)**	**No. of transmembrane domain**
orf124a	+	48069	48443	0
orf119a-1	+	30788	31147	1
orf111a-1	-	14252	13917	0
orf652a	+	118311	120266	1
orf220a	-	115742	115083	0
orf108a	-	117068	116745	0
orf186a	+	206564	207121	1
orf114a	+	318544	318885	1
orf104a	+	315276	315587	0
orf348a	-	353406	352363	2
orf185a	+	398641	399195	0
orf169a	+	353039	353545	0
orf164a	+	398298	398789	1
orf128a	+	427161	427544	0
orf289a	-	495484	494618	0
orf119a-2	-	485721	485365	1
orf118a	+	575607	575960	2
orf115a	+	46226	46573	0
orf154a	+	99164	99625	0
orf102a	+	98613	98918	0
orf142a	+	144268	144693	2
orf263a	+	190047	190835	0
orf160a	-	232803	232324	2
orf178a	-	291952	291419	0
orf161a	+	294127	294609	0
orf110a	-	273472	273143	0
orf111a-2	-	595914	595582	0
orf157a	-	98264	97794	1
orf399a	+	145547	146743	10
orf314a	-	514127	513186	3

Table S6 The open reading frames (ORFs) more than 100 amino acids in SI3A mitochondrial genome

Gene ID	strand	mRNA start(bp)	mRNA stop(bp)	No. of transmembrane domain
orf160a	-	1345	863	0
orf158a	-	564	88	0
orf162a	-	2411	1923	3
orf121a	+	4220	4585	0
orf119a-1	-	37957	37598	1
orf108a-1	+	2596	2922	0
orf107a-1	+	3276	3599	0
orf124a-1	-	20595	20221	0
orf129a	+	87904	88290	0
orf120a	+	102539	102898	0
orf109a	-	116574	116248	0
orf119a-2	-	197931	197575	1
orf111a-1	+	214477	214809	0
orf131a	+	48253	48648	0
orf276a	+	149169	149999	0
orf190a	+	117057	117626	0
orf108a-2	-	147919	147596	0
orf159a	+	23773	24252	2
orf132a	-	45811	45413	1
orf606a	+	149166	150998	1
orf212a	-	15842	15204	0
orf112a	-	24209	23871	0
orf101a	+	60615	60917	0
orf106a	-	107535	107218	0
orf135a	-	243079	242675	0
orf123a	-	215867	215499	2
orf102a-1	+	228084	228389	0
orf102a-2	-	225144	224839	0
orf111a-2	+	321675	322007	0
orf117a	+	356916	357266	0
orf107a-2	+	356266	356586	0

orf124a-2	-	89479	89108	0
orf317a	+	148101	149051	3
orf118a	+	172377	172730	0
orf102a-3	-	167689	167384	0
orf157a	-	195280	194810	1
orf382a	+	298851	299999	9
orf114a	+	6899	7243	1
